# Cochleaimplantatversorgung bei Autoimmunschwerhörigkeit

**DOI:** 10.1007/s00106-024-01472-5

**Published:** 2024-04-22

**Authors:** Maximilian Armstorfer, Lennart Weitgasser, Stefan Tschani, Sebastian Rösch

**Affiliations:** 1 Klinik für Hals-Nasen-Ohren-Heilkunde, Universitätsklinikum Salzburg, Müllner Hauptstraße 48, 5020 Salzburg, Österreich; 2 Klinik und Poliklinik für Hals-Nasen-Ohren-Heilkunde, Universitätsklinikum Regensburg, Regensburg, Deutschland

**Keywords:** Hörhilfen, Prothesen und Implantate, Monoklonale Antikörper, Tumornekrosefaktorinhibitoren, Adalimumab, Hearings aids, Prostheses and implants, Monoclonal antibodies, Tumor necrosis factor inhibitors, Adalimumab

## Abstract

**Hintergrund:**

Die Autoimmun-Innenohrerkrankung („autoimmune inner ear disease“, AIED) imponiert durch rezidivierende, fluktuierende sensorineurale Schwerhörigkeit mit assoziierten vestibulären Symptomen. Therapeutisch kommen Kortikosteroide und Immunsuppressiva zum Einsatz. Trotz Behandlung kann es zu progressiver Schwerhörigkeit bis hin zur Ertaubung kommen, sodass ein Cochleaimplantat (CI) indiziert ist. Hier wird der Fall eines 25-jährigen Patienten präsentiert, bei dem es im Rahmen der CI-Versorgung zu Impedanzschwankungen kam, welche in Abhängigkeit von der vorab begonnenen Therapie mit dem Tumornekrosefaktor(TNF)-α-Inhibitor Adalimumab standen.

**Zielsetzung:**

Hat eine immunmodulatorische Therapie bei AIED-Patienten nach CI-Versorgung einen Einfluss auf die Versorgungsqualität?

**Material und Methoden:**

Die Autoren dokumentierten über ein Jahr hinweg die Impedanzen und die Ergebnisse des Freiburger Sprachtests ihres Patienten in Abhängigkeit von der Adalimumab-Therapie.

**Ergebnisse:**

Unmittelbar nach Implantation zeigten sich unauffällige Impedanzwerte. Im weiteren Verlauf nahmen diese jedoch zu, weshalb rezidivierend Anpassungen der CI-Funktion nötig waren. Nach Wiederbeginn der Therapie mit Adalimumab sanken die Impedanzen wieder ab.

**Schlussfolgerung:**

Eine CI-Versorgung kann bei AIED-Patienten eine suffiziente Hörrehabilitation ermöglichen. Je nach Aktivität der Grunderkrankung kann es jedoch zu schwankenden Impedanzen kommen. Eine immunmodulatorische Therapie kann somit durchaus sinnvoll und nötig sein, um eine adäquate Hörrehabilitation mit dem CI zu ermöglichen.

## Abstract (en)

**Background:**

Autoimmune inner ear disease (AIED) manifests with recurrent fluctuating sensorineural hearing loss and vestibular symptoms. Treatment includes steroids and a variety of immunosuppressants. Despite adequate treatment, sensorineural hearing loss can be progressive to the point of deafness. In these patients, a cochlear implant (CI) is indicated. We present the case of a 25-year-old male who underwent cochlear implantation in the left ear. After implantation we noticed brisk variations in impedances which were related to application of the previously prescribed tumor necrosis alpha (TNFα) inhibitor adalimumab.

**Objective:**

Can immunomodulatory therapy in AIED patients after CI fitting affect the quality of hearing rehabilitation?

**Materials and methods:**

We documented impedances and speech intelligibility (Freiburg monosyllable test) of our patient for 1 year in dependence on adalimumab therapy.

**Results:**

Directly after implantation, impedances were within the normal range. During the further course, impedances started to rise, and recurrent adjustments of the implant were needed. Adalimumab therapy was reinitiated, which resulted in a subsequent reduction of impedances.

**Conclusion:**

Cochlear implantation can be necessary in some AIED patients and poses a sufficient method for hearing rehabilitation. Depending on the activity of the underlying disease, a rise in impedances may occur. Immunomodulatory treatment may therefore be necessary to maintain adequate hearing results with the CI.

## Einleitung

Die Autoimmun-Innenohrerkrankung („autoimmune inner ear disease“; AIED) wurde 1979 als eigene Entität beschrieben [1]. Sie ist charakterisiert durch einen bilateralen, sensorineuralen Hörverlust. Dieser ist häufig primär steroidsensibel, im Verlauf entwickelt sich jedoch bei den meisten Patienten eine Steroidresistenz. Vestibuläre Symptome können in verschiedenen Ausprägungen vorliegen, weitere Organmanifestationen treten nicht auf. Davon abzugrenzen ist die sekundäre AIED, welche im Rahmen anderer Autoimmunerkrankungen auftritt [2].

Die Diagnose wird aufgrund des klinischen Verlaufs gestellt. Verschiedene Laborparameter wurden beschrieben, einheitliche Diagnosekriterien bestehen jedoch zum aktuellen Zeitpunkt nicht. Ein möglicher diagnostischer Algorithmus wurde 2020 publiziert. Wegweisend sind die Klinik und die audiovestibulären Befunde. Mittels Magnetresonanztomographie (MRT) sowie laborchemischer Untersuchungen in Kooperation mit einer rheumatologischen Klinik sollten andere Differenzialdiagnosen abgegrenzt werden [3].

## Therapeutische Ansätze

Therapeutisch stehen verschiedene Ansätze zur Verfügung. Primär sollte ein Behandlungsversuch mit einem systemischen Steroid versucht werden. Bei ausbleibender Besserung kann eine intratympanale Steroidtherapie versucht werden. Bei vielen Patienten kommt es zu einer initialen Besserung, zumeist entwickelt sich jedoch im Verlauf eine Steroidresistenz [3]. In weiterer Folge stehen verschiedene Biologika zur Verfügung. Die am häufigsten angewendeten Präparate blockieren die Funktion von Interleukin(IL)-1, TNF‑α oder CD20. [3–7].

Unter diesen Therapien stabilisiert sich oftmals die Symptomatik, jedoch kann ein Fortschreiten nicht in jedem Fall verhindert werden. In diesem Fall bleibt oftmals als einzige Option der Hörrehabilitation ein CI übrig [8]. Im Folgenden wird über den Krankheitsverlauf eines jungen Patienten mit AIED ab dem Zeitpunkt der CI-Implantation berichtet.

## Fallvorstellung

### Anamnese

**Abb. 1:**
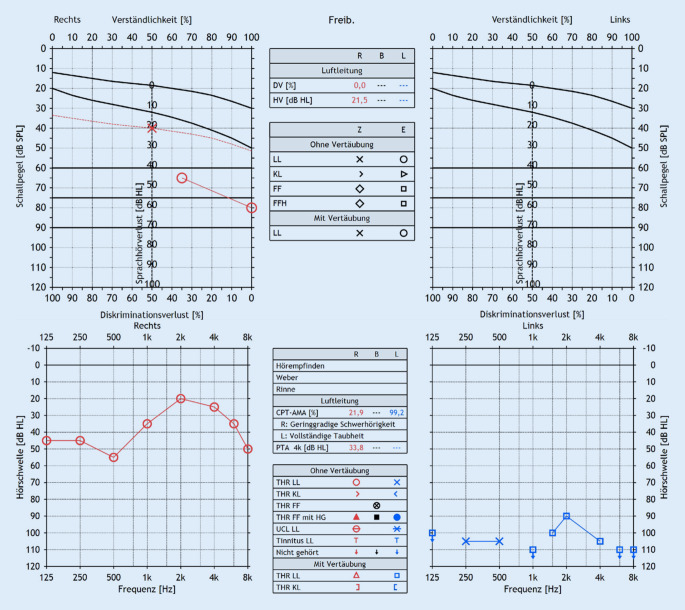
Ton- und Sprachaudiogramm vor der Implantation (September 2022)

Vorgestellt wird der Fall eines 25-jährigen männlichen Patienten, der mit einer linksseitig beginnenden, in der Folge asymmetrischen, beidseitigen, fluktuierenden sensorineuralen Schwerhörigkeit mit Drehschwindel und Tinnitus vorstellig wurde. Auf eine systemische Steroidtherapie trat eine kurzfristige Besserung auf, wenige Wochen darauf kam es aber zu einer Progredienz. Weitere Therapieversuche mit Steroiden – intravenös und intratympanal – erbrachten keine Besserung. Nach Ausschluss anderer Differenzialdiagnosen sowie bei beidseitigen fluktuierenden Hörschwellen und Nachweis einer beidseitigen vestibulären Unterfunktion mittels pathologischem Video-Kopfimpulstest und kalorischer Testung, stellte sich die Verdachtsdiagnose einer AIED. Zur Differenzialdiagnostik erfolgten eine serologische Erregerdiagnostik, Serumelektrophorese, Hormonessay, rheumatologische Diagnostik sowie Routinelabordiagnostik, welche keine wegweisenden Ergebnisse erbrachten. Die Hirnstammaudiometrie zeigte weder eine auditorische Synaptopathie noch eine retrocochleäre Hörstörung. In der MRT zeigte sich eine leicht reduzierte Flüssigkeitsmarkierung des Labyrinths beidseits. Es erfolgte eine initiale orale Therapie mit Prednisolon (40 mg/Tag) über mehrere Monate. Bei persistierender Symptomatik wurde diese beendet. Es erfolgte eine Plasmapherese, welche nur zu einer kurzfristigen Besserung führte. Ein Therapieversuch mit dem IL-1-Antagonisten Anakinra s.c. führte zu einer Stabilisierung der Hörschwelle. Folglich wurde die Therapie auf den TNF-α-Antagonisten Adalimumab umgestellt, da die s.c.-Applikation alle 14 Tage gegenüber täglicher Applikation anwendungsfreundlicher für den Patienten war. Die Therapie erfolgte in rheumatologischer Absprache. Für den detaillierten Verlauf der Abklärung sowie die bisherigen Therapien wird auf die Vorpublikation verwiesen [9]. Im Verlauf kam es links zu einer Verschlechterung der Hörschwelle, weshalb im September 2022 der Entschluss zur Cochleaimplantation links gefasst wurde. Die Abb. 1 zeigt das Tonschwellen- und Sprachaudiogramm vor der Implantation. Es erfolge die Implantation eines Cochlear-Nucleus-Geräts (Fa. Cochlear, Hannover, Deutschland; CI-622-Elektrode). Mit der antiinflammatorischen Therapie wurde perioperativ pausiert, und bei vorerst stabilen Befunden und Symptomen wurde sie bis auf Weiteres ausgesetzt. Im Rahmen der Nachsorge erfolgten regelmäßige Kontrollen der Impedanzen mittels der vom Hersteller zur Verfügung gestellten Software. Die Sprachverständlichkeit wurde mittels Freiburger Sprachtest aufgezeichnet. Diese Parameter wurden retrospektiv mit den Adalimumab-Applikationen auf einer Zeitachse dargestellt.

### CI-Impedanzmessung

**Abb. 2:**
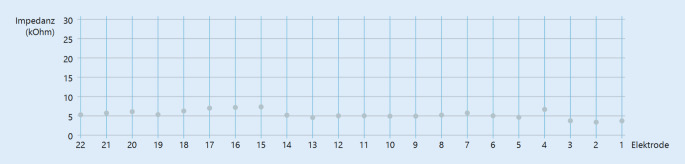
Impedanzen bei Erstanpassung (September 2022)

Bei Erstanpassung zeigten sich die Impedanzen zwischen 3,9 und 8,4 kOhm bei einem Zielbereich von 0,5–20 kOhm (Abb. 2).

**Abb. 3:**
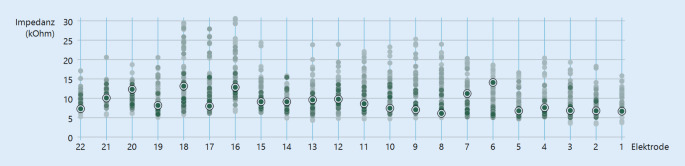
Impedanzen sämtlicher Messungen (*n* = 56) von September 2022 bis November 2023. Rezenteste Messungen hervorgehoben, je *heller* die Messpunkte, desto länger zurückliegend

Im Verlauf kam es zu einer Zunahme und zu Schwankungen der Impedanzen (Abb. 3). Am ausgeprägtesten zeigten sich diese bei den Elektroden 16–18. Hier wurden Impedanzen von 6,1 bis > 30 kOhm (Mittelwert = 12,9) gemessen, wobei 30 kOhm die maximale Messschwelle der Software darstellt. Subjektiv berichtete auch der Patient über schwankendes Sprachverstehen bei Verwendung des CIs.

Es erfolgte zum Ausschluss einer Labyrinthitis ossificans und zur Elektrodenlagekontrolle eine Computertomographie, die einen regelrechten Befund ergab.

Somit wurde die antiinflammatorische Therapie mit dem TNF-Alpha-Antagonisten Adalimumab wieder begonnen. Ab März 2023 wurden 40 mg Adalimumab in 14-tägigen Intervallen appliziert, ab April 80 mg alle 14 Tage.

**Abb. 4:**
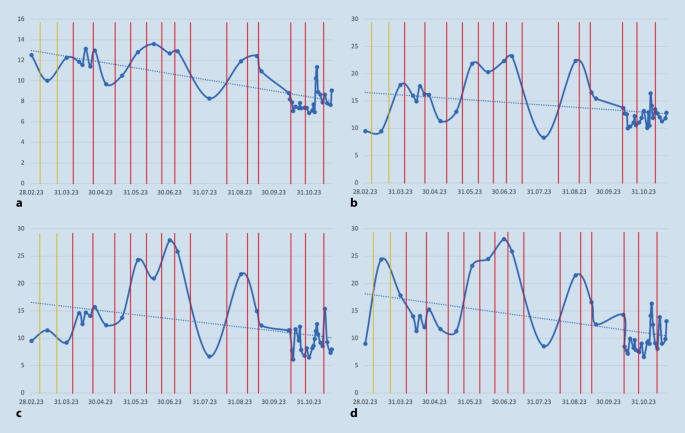
Durchschnittliche Impedanzen sowie die Impedanzen der am meisten betroffenen Elektroden 16–18 in kOhm (*y‑Achse*) über die Zeit (*x‑Achse*). *Linien *Adalimumab-Applikationen (*gelb* 40 mg, *rot* 80 mg). Trendlinie (*gestrichelte Linie*): insgesamt fallende Impedanzen über den Messzeitraum

**Abb. 5:**
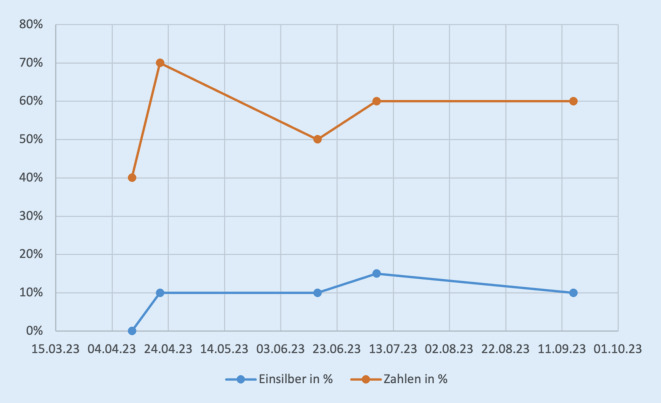
Ergebnisse der Sprachaudiometrie im Verlauf

Es zeigte sich eine Stabilisierung und Abnahme der Impedanzen (Abb. 4). Messungen erfolgten anfangs monatlich, in der Folge beinahe täglich. Sprachaudiometrien wurden regelmäßig durchgeführt. Hier zeigten sich nach Beginn der Therapie stabile Befunde. Insgesamt wurde jedoch nur eine geringe Sprachverständlichkeit zwischen 10 und 15 % Einsilberverständnis erreicht, während das Zahlenverstehen von 40 auf 60 % stieg (Abb. 5).

## Diskussion

Eine CI-Versorgung ist bei AIED-Patienten eine relevante Therapieoption. Die Nachsorge birgt jedoch Herausforderungen. Fluktuationen der Hörwahrnehmungen sind möglich. Impedanzschwankungen bei CI-versorgten AIED-Patienten wurden bereits beobachtet [10]. Die Autoren vermuten, dass die fortwährende Entzündungsreaktion hierfür ursächlich ist. Dies würde erklären, warum unter immunmodulatorischer Therapie eine Stabilisierung der Impedanzen auftritt. Zudem berichtete der hier vorgestellte Patient über geringere Fluktuationen in der Hörwahrnehmung. Gemäß Literatur sind bei CI-Versorgung von AIED-Patienten gute Ergebnisse zu erwarten [8]. Die Freiburger Sprachtests im vorliegenden Fall zeigten dennoch nur geringe Werte für Einsilberverständlichkeit (Abb. 5). Bei der Zahlenverständlichkeit konnten höhere Werte erreicht werden. Hier zeigt sich auch eine Korrelation aus den niedrigsten Verständlichkeitswerten zu den höchsten Impedanzwerten im Juni 2023 (Abb. 4 und 5).

Es ist anzumerken, dass es vor Einleitung der Adalimumab-Therapie zudem zu starken subjektiven Schwankungen in der Sprachverständlichkeit kam, welche für den Patienten eine Belastung darstellten. Subjektiv besserten sich diese, dies zeigt sich in den audiometrischen Daten jedoch nicht, da diese hierfür nicht regelmäßig genug geprüft wurden. Erklärend für das schlechte Sprachverstehen sind zudem hohe Pulsbreiten, die aufgrund der Impedanzen notwendig waren. Zudem erschwerten die Schwankungen die Anpassung erheblich. Eine endgültige Erklärung für die schlechten Ergebnisse besteht jedoch Autoimmun-Innenohrerkrankung („autoimmune inner ear disease“, AIED) nicht.

Zusammenfassend lässt sich sagen, dass trotz CI-Versorgung auf eine immunmodulatorische Therapie nicht immer verzichtet werden kann.

Die Schwindelbeschwerden änderten sich unter keiner der durchgeführten Therapiemodalitäten, stehen für den Patienten jedoch zum aktuellen Zeitpunkt nicht im Vordergrund.

## Fazit für die Praxis

- Eine Cochleaimplantat(CI)-Versorgung für Patienten mit Autoimmun-Innenohrerkrankung („autoimmune inner ear disease“, AIED) ist möglich und in Einzelfällen sinnvoll.
- Aufgrund der Möglichkeit einer cochleären Obliteration sollte diese rasch erfolgen.
- Patienten sollten über eine eventuell notwendige fortwährende immunmodulatorische Therapie aufgeklärt werden.
- Aufgrund möglicher Impedanzschwankungen sind regelmäßige Anpassungstermine nötig.
- Hiermit verbunden kann u. U. ein schlechteres Ergebnis bei der CI-Versorgung resultieren.
